# Utilizing XGBoosts to correct arcjet contamination in magnetic field measurements from GOES missions

**DOI:** 10.3389/frai.2025.1628029

**Published:** 2025-09-16

**Authors:** Fadil Inceoglu, Paul T. M. Loto'aniu

**Affiliations:** ^1^Cooperative Institute for Research in Environmental Sciences, University of Colorado Boulder, Boulder, CO, United States; ^2^National Centers for Environmental Information, National Oceanic and Atmospheric Administration, Boulder, CO, United States

**Keywords:** machine learning, XGBoost, arcjet, GOES, magnetic field

## Abstract

The magnetometers onboard the Geostationary Operational Environmental Satellites (GOES) provide crucial measurements for space weather monitoring and scientific research. However, periodic arcjet thruster firings introduce contamination in the measured magnetic field, affecting data accuracy. The currently used correction matrix approach mitigates these effects but struggles with transient variations and residual errors. In this study, we present an alternative correction method using XGBoost, a machine learning algorithm, to correct arcjet-induced contamination in the GOES-17 magnetometer data using GOES-18 as ground truth. Using cross-satellite comparisons and supervised learning techniques, our model is effective in reducing artificial disturbances, especially non-linear variations. We found that the XGBoost method works better than the existing correction matrix approach for E and P components, while the correction matrix performs better for the N component. Although some limitations remain due to training data constraints, our results highlight the importance of machine learning to improve magnetometer data quality by recognizing and correcting complex satellite-driven artifacts. The collocation of GOES-17 and GOES-18 provided a unique opportunity for cross-satellite calibration and validation, and with a longer collocation period, the XGBoost method shows significant promise for better correction of operational data, emphasizing the need for such configurations in future satellite missions.

## 1 Introduction

Geostationary Operational Environmental Satellites (GOES) have continuously measured Earth's magnetic field from geostationary orbit for more than 40 years, providing critical data for space weather monitoring and scientific research. The magnetometer (MAG) instruments onboard these satellites are operationally used by NOAA's Space Weather Prediction Center (SWPC) for real-time space weather forecasting and event detection, including geomagnetic storms, radiation belt dynamics, and magnetospheric processes (Loto'aniu et al., 2023). Beyond operational use, GOES magnetometer data serve as a fundamental resource for space physics research, playing a key role in empirical magnetospheric modeling and the study of ultralow-frequency (ULF) waves (Loto'aniu and Inceoglu, 2024).

The GOES-R series magnetometer measurements are periodically contaminated by arcjet thruster firings, which are performed to maintain spacecraft position–specifically for North-South station keeping–in geostationary orbit. Arcjet thrusters generate thrust by electrically heating hydrazine propellant, producing a partially ionized plasma plume whose elevated density and plasma pressure cause local magnetic field disturbances near the spacecraft. These firings introduce magnetic field perturbations, primarily due to the diamagnetic effect of the thruster plume and pressure gradients in the surrounding plasma environment (Califf et al., 2020a,b). Previous studies have highlighted the need for correction methods, as arcjet disturbances can introduce biases in the measured magnetic field data (Loto'aniu et al., 2023). Califf et al. (2020b) developed the currently used correction matrix approach that applies linear adjustments to mitigate the effects of arcjet contamination, but this method has limitations in handling transient variations and residual errors in corrected data.

Machine learning methods have recently been explored to improve the quality of magnetometer data. Inceoglu and Loto'aniu (2021) applied both supervised and unsupervised learning to correct offset anomalies stemming from thermal and seasonal effects in the GOES-16 magnetometer data, demonstrating that machine learning models can adapt to complex and non-linear variations in spacecraft magnetic field measurements. Building on this broader interest in ML-based correction methods, we explore the use of XGBoost (Chen and Guestrin, 2016), a tree-based gradient-boosting machine learning model, to correct arcjet-induced contamination in GOES-17 data using collocated GOES-18 magnetometer data as ground truth. Using supervised learning techniques and cross-satellite comparisons, we aim to develop a robust correction model that adapts to the dynamic characteristics of arcjet disturbances. GOES-18, which benefits from improved thermal stability and instrument design compared to GOES-16 and GOES-17 (Loto'aniu et al., 2023), provides an ideal dataset to be used as the ground truth for training and validating the correction algorithm.

## 2 Data and methods

### 2.1 Overview of the GOES-R series and magnetometer design

The GOES-R series (GOES-16 to GOES-19) represents the most recent generation of NOAA's geostationary satellites, designed for continuous weather and space weather monitoring. GOES-16 (launched in November 2016, earlier GOES-East) is located at 75.2°W. GOES-17 (launched in March 2018) served as GOES-West at 137.2° W from February 2019 until January 2023 (Loto'aniu et al., 2023), after which it was relocated to on-orbit storage at 105° W.^1^ GOES-18 (launched in March 2022) transitioned to 137.0° W in mid-2022 and fully assumed operational GOES-West status by early January 2023 (Loto'aniu et al., 2023). Each spacecraft carries a pair of fluxgate magnetometers mounted on a boom, with the inboard (IB) sensor positioned 6.3 m and the outboard (OB) sensor 8.5 m from the spacecraft. The IB sensor is more susceptible to thermal influences from the spacecraft bus, whereas the OB sensor generally provides greater long-term stability (Loto'aniu et al., 2023).

Following its launch in March 2022, GOES-18 was positioned close to GOES-17 for 2.5 months, with a longitudinal separation of only 0.4 degree (136.8°W vs. 137.2°W). This first of its kind collocation provided a unique opportunity for direct cross-satellite calibration, as both spacecraft observed nearly identical geomagnetic conditions. These comparisons demonstrated that GOES-18, equipped with the new Goddard magnetometers (GMAG), exhibits diurnal and long-term stability within ±1 nT, compared to variations of ~2 nT on GOES-17 and >10 nT on GOES-16 (Loto'aniu et al., 2023). This improvement reflects both the change in vendor (NASA Goddard vs. MEDA for GOES-16/17) and significant engineering upgrades, including redesigned sensor and electronics units, added heaters and thermal isolation spacers, enhanced blanketing, and extensive ground and on-orbit thermal testing, all of which mitigated the thermal instabilities seen in earlier MAG instruments (Loto'aniu et al., 2023).

### 2.2 Contamination in the magnetic field measurements

**Figure 1:**
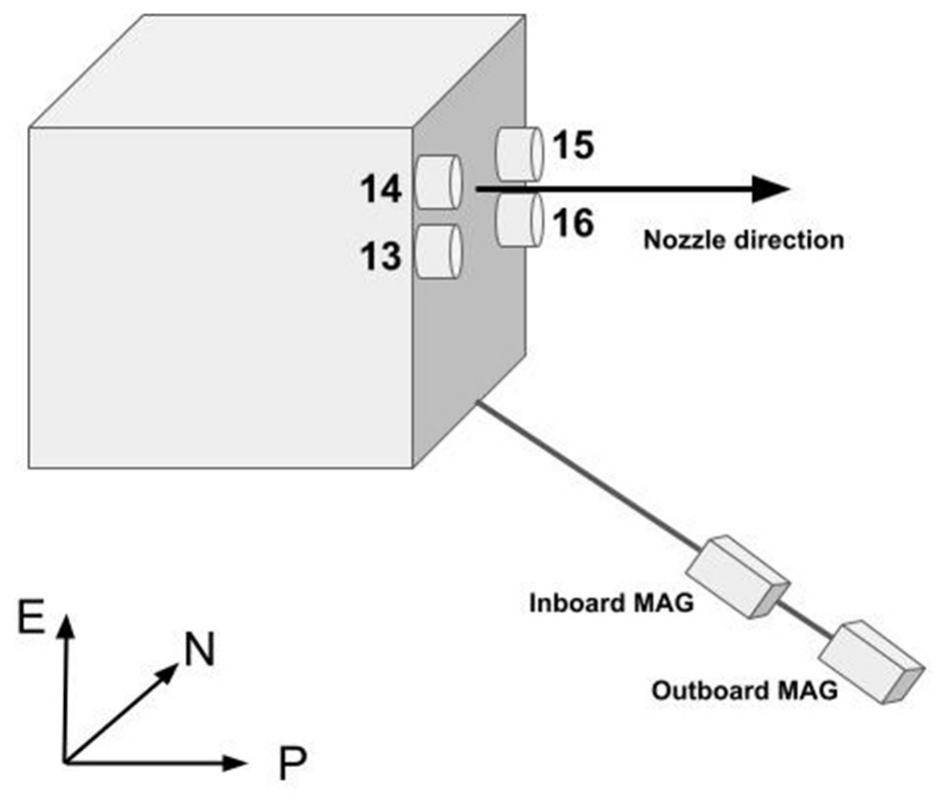
Illustration of the inboard and outboard GOES magnetometers mounted on a boom and arcjet thrusters. The axes show the relationship between earthward-poleward-normal (EPN) coordinate frame (Califf et al., 2020a).

The arcjet thrusters aboard the GOES-R series satellites—GOES-16 to GOES-19—are periodically fired to maintain the spacecraft's geostationary orbit. Each satellite is equipped with four thrusters, numbered 13, 14, 15, and 16 (Figure 1), nominally fire in alternating pairs (13–15 or 14–16) approximately every four days for around 90 minutes per maneuver (Califf et al., 2020a), although the exact pairing may occasionally vary. During these firings, the GOES-R magnetometers detect a significant artificial disturbance in the local magnetic field, introducing deviations of up to ~20 nT, 20% of the typical geomagnetic field strength at geostationary orbit (Califf et al., 2020b). This contamination occurs due to two primary physical mechanisms: (i) the diamagnetic effect of the thruster plume, which reduces the local ambient magnetic field strength, and (ii) plasma pressure gradients within the thruster exhaust that introduce additional localized magnetic perturbations (Califf et al., 2020b,a). The disturbance is most pronounced along the thrust axis, primarily affecting the poleward (P) component of the measured magnetic field, and remains relatively stable throughout the burn period. Although rapid recovery of the magnetic field occurs once the thrusters are deactivated, small residual offsets (~1-2 nT) may persist for hours due to thermal effects on the magnetometer electronics (Califf et al., 2020a).

**Figure 2:**
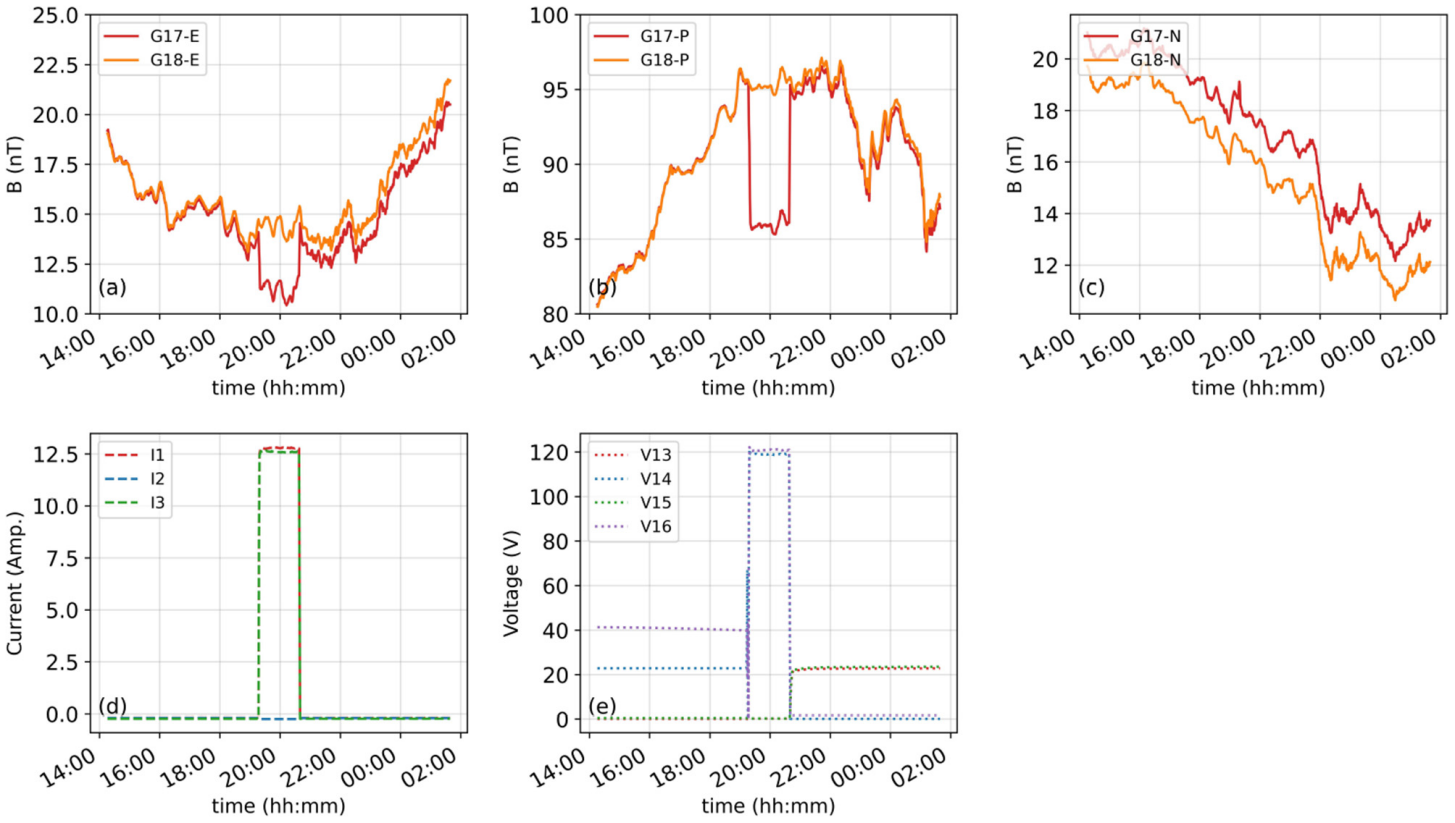
The top panels show the magnetic field measurements from 26-27 July 2022 from GOES-17 (red) and GOES-18 (orange) in the E **(a)**, P **(b)**, and N **(c)** components of the EPN frame. The lower panels **(d, e)** show the current and voltage values corresponding to the arcjet firings on GOES-17.

Similar contamination in magnetic field measurements can also be observed in GOES-17 and GOES-18 (Figure 2). During the GOES-17/18 collocation described above, both satellites measured the Earth's magnetic field under nearly identical conditions, enabling direct cross-satellite comparisons. Outside this overlap, the longitudinal separation between the satellites leads to differences in the ambient field, preventing one from being used as ground truth for the other.

During the collocation, we identified 21 GOES-17 arcjet firing events during which GOES-18 did not fire its arcjets simultaneously. This situation provided us with a great opportunity to directly compare the effects of arcjet contamination in GOES-17 measurements. To avoid ambiguity, only events that occurred during geomagnetically quiet to moderate conditions (generally Kp ≤ 4, with no major disturbances such as magnetopause crossings) were selected. Any background wave activity present during the collocation would have been observed nearly identically by both satellites (given their 0.4 degree longitudinal separation) and therefore does not introduce bias in the cross-satellite correction. All magnetic field data are analyzed in the Earth-Pointing Normal (EPN) coordinate system, where the X-axis points toward Earth's center, the Y-axis is aligned opposite the solar panel axis (approximately anti-sunward), and the Z-axis completes the right-handed system, generally pointing northward along the spacecraft's orbital normal. This coordinate system is spacecraft-fixed and provides a stable reference frame for interpreting arcjet-related disturbances, which are closely aligned with specific spacecraft structures and thruster directions. When the arcjets are activated, their current (Figure 2c) and voltage (Figure 2d) levels increase and remain elevated while firing. Simultaneously, there is a clear reduction in the magnetic field measurements in the E and P components of the EPN coordinate frame, which persists for the full firing duration (Figures 2a, b).

Given the significance of GOES magnetometer data for scientific research, mitigation of arcjet-induced contamination is crucial. A correction algorithm was developed for both the OB and IB magnetometers on each GOES-R series spacecraft, based on an observed linear relationship between the arcjet disturbance and the ambient magnetic field (Califf et al., 2020a). While both sensors are corrected independently, OB data are generally prioritized due to its improved thermal and bias stability. This least-squares regression correction was initially found to reduce residual errors to below 1.5 nT during steady-state firings in GOES-16 (Califf et al., 2020a). However, in cases involving non-nominal firing configurations, such as single-thruster firings or evolving thrust levels, residuals from the matrix correction can be substantially larger, particularly on GOES-17 (Califf et al., 2020a). These abnormal cases are relatively uncommon compared to the standard paired-thruster maneuvers but illustrate the need for more robust correction techniques. Our study focused on well-defined paired thruster firings during the GOES-17/18 collocation; therefore, the performance of XGBoost in these rarer abnormal cases remains to be evaluated. Importantly, the existing correction matrix does not account for the short-lived transient effects at the start and end of arcjet burns, which are flagged as invalid in operational data. In designing our approach, we developed the XGBoost model to better capture these transient shoulders, providing a framework that can in principle address such short-duration features.

### 2.3 XGBoost: training, validation, and test

XGBoost (Extreme Gradient Boosting) is an advanced, scalable tree boosting system designed for efficiency, accuracy, and performance in large-scale machine learning applications (Chen and Guestrin, 2016). It is an implementation of gradient boosted decision trees (Friedman, 2001) that introduces several optimizations to enhance speed and scalability. XGBoost iteratively constructs an ensemble of decision trees, where each new tree corrects errors made by previous trees, optimizing an objective function through gradient descent (Chen and Guestrin, 2016). The system incorporates a regularized learning objective to control model complexity and mitigate overfitting, ensuring generalization to unseen data.

To develop XGBoost models for each EPN coordinate frame component, we used data from the GOES-17/18 collocation period. We selected 21 GOES-17 arcjet firing events during which GOES-18 had no simultaneous firings, ensuring a clean reference for training and validation. GOES-18 magnetic field measurements were used as the ground truth, and data from both IB and OB magnetometers were combined to increase the training volume and enhance generalizability.

**Figure 3:**
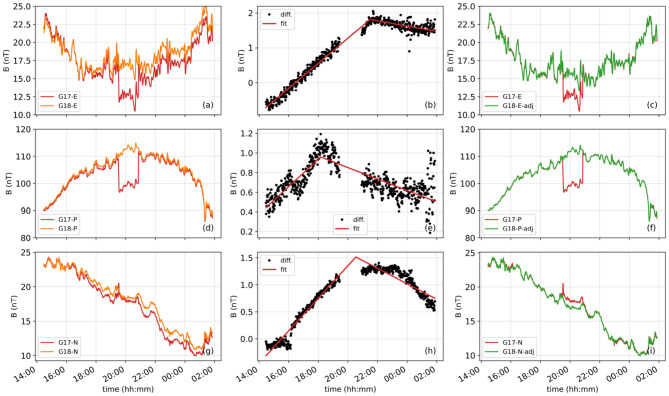
The left panels **(a, d, g)** show the magnetic field measurements from GOES-17 (red) and GOES-18 (orange) in the E **(a)**, P **(d)**, and N **(g)** components of the EPN coordinate frame. The middle panels **(b, e, h)** show the differences between the GOES-18 and GOES-17 magnetic field measurements (black dots) for the E **(b)**, P **(e)**, and N **(h)** components, together with the piecewise linear regression fits (red lines). The right panels **(c, f, i)** show the adjusted GOES-18 values (green) after subtracting the piecewise linear regression fits for the E **(c)**, P **(f)**, and N **(i)** components.

Before training the XGBoost models, we preprocessed the data to make sure that the GOES-18 data, which is used as the ground truth, and the GOES-17 data, which will be corrected, overlap without any longer-term effects due to the slightly higher thermal sensitivity of the GOES-17 magnetometers (Figure 3). To achieve this objective, we first subtracted the GOES-18 measurements in each component of the EPN coordinate frame from the GOES-17 measurements during the periods when there are no arcjet firings (Figures 3a, d) for each of the 21 days from the IB and OB magnetic field data. Subsequently, we fitted a piecewise linear regression function to each difference to determine the longer-term difference between the two satellites (Figures 3b, e). We then subtracted this longer-term trend in magnetic field measurements from the GOES-18 data for each component in the EPN frame (Figures 3c, f) to create “adjusted GOES-18” values that effectively represent what GOES-17 would have measured in the absence of arcjet contamination. These adjusted values are no longer the raw GOES-18 measurements but serve as a proxy ground truth for training and validation.

Training, validation, and test sets were created from 21 GOES-17 arcjet events during the collocation period, using adjusted GOES-18 data as ground truth and GOES-17 current and voltage as inputs. We restricted the inputs to spacecraft-intrinsic parameters (current and voltage), as these directly drive the contamination mechanism and keep the correction algorithm self-contained. External variables such as the ambient magnetic field or interplanetary conditions were not included, because GOES satellites are normally located at different longitudes and the 2.5-month collocation period did not provide enough variability in interplanetary and ambient magnetic fields to train a generalized model that incorporates broader space weather effects.

Each “dataset” refers to a time window centered on a single arcjet firing, typically spanning the duration of the maneuver with a buffer before and after. This process yielded 30 arcjet events across 15 days for training, 4 events from 2 days for validation, and 4 events from 4 days for testing. Only IB magnetometer data were used for testing, as IB and OB arcjet responses are highly similar. To ensure test-day independence, all OB data from the test days were excluded from the training and validation sets.

Hyperparameter optimization was explored using a Bayesian search over a range of values (Table 1). However, optimization did not yield significant improvements compared to the default values, largely due to the limited size and variability of the training dataset. Therefore, we retained the default values in all further analyses.

**Table 1:** Default XGBoost hyperparameter values and the parameter ranges explored using Bayesian optimization.

**Parameter**	**Default value**	**Search range**
booster	gbtree	gbtree
learning_rate	0.3	{0.0001, 0.001}
max_depth	6	{64, 128, 256}
max_leaves	-	{128, 256, 512}
n_estimators	100	{30, 100, 500, 1,000, 1,500}
max_bin	-	{10, 100, 1,000}
min_child_weight	-	[0.01, 10.0]
subsample	1	-
colsample_bytree	1	-
gamma	0	-
reg_alpha	0	-
reg_lambda	1	-

Parameters without a search range were kept fixed.

## 3 Results

The R^2^ and mean squared error (MSE) values for each component of the EPN coordinate frame for the training and validation sets display very high and low values, respectively (Table 2). These values primarily reflect the fact that the GOES-17 data and the adjusted GOES-18 ground truth are nearly identical outside the arcjet firing periods, which make up roughly 10% of the total data length. Since the XGBoost correction algorithm is applied across the full time window–including before, during, and after arcjet events–the evaluation metrics are computed over the entire period to reflect overall model performance. Visual inspection of the corrected time series during arcjet activity is used in tandem to assess the model's effectiveness in addressing the contamination.

**Table 2:** XGBoost performance metrics for training and validation sets.

	**Training**		**Validation**	
	**R** ^2^	**MSE**	**R** ^2^	**MSE**
**E**	0.9983	0.1066	0.9897	0.1734
**P**	0.9999	0.0598	0.9977	0.4944
**N**	0.9993	0.0317	0.9903	0.1101

We chose four dates as our test days where GOES-17 fired its arcjets to maneuver while GOES-18 continued to measure the undisturbed magnetic field; 22, 26, and 29 July and 2 August 2022. Using the corrected IB magnetic field data from GOES-17 and the adjusted magnetic field data from GOES-18 we calculated the Pearson correlations and MSE values. The results show that our correction algorithm for each component of the EPN coordinate frame has very strong correlations, except for the component P on August 2nd, and component E on July 29th when the R^2^ is lower and hence the MSE value is higher compared with other days and components (Table 3).

**Table 3:** Correlation coefficients and mean squared errors (MSE) for E, P, and N components calculated using the corrected GOES-17 magnetic field data and adjusted GOES-18 data for the test set.

**Date**	**E**		**P**		**N**	
	**R** ^2^	**MSE**	**R** ^2^	**MSE**	**R** ^2^	**MSE**
22 July	0.9713	0.3612	0.9976	0.2419	0.9973	0.1058
26 July	0.9887	0.0991	0.9985	0.0711	0.9903	0.2333
29 July	0.9559	0.2746	0.9980	0.2217	0.9970	0.0424
02 Aug	0.9938	0.1258	0.9774	2.7067	0.9966	0.1766

**Figure 4:**
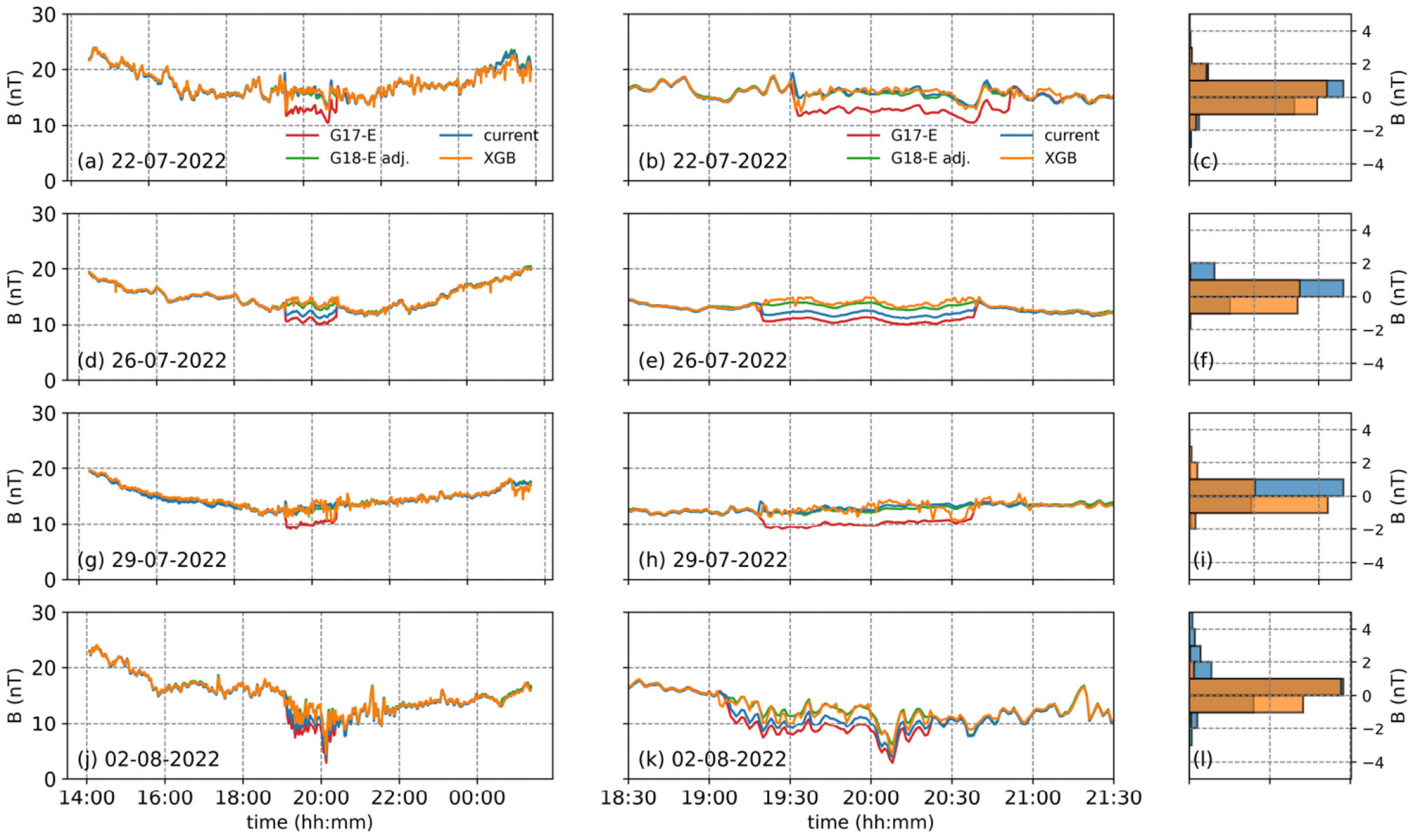
The left panel shows 4 dates we tested the XGBoost developed to correct the E component of the EPN coordinate frame from GOES-17 (red), adjusted GOES-18 (green), the corrected GOES-17 using the current (blue) and XGBoost (orange) methods. The middle panel shows the same but focused around the arcjet firing periods, while the right panel shows the histograms of the differences between the adjusted GOES-18 values and the XGBoost (orange) and the current (blue) models. The left panel **(a, d, g, j)** shows 4 dates we tested the XGBoost developed to correct the E component of the EPN coordinate frame from GOES-17 (red), adjusted GOES-18 (green), the corrected GOES-17 using the current (blue) and XGBoost (orange) methods. The middle panel **(b, e, h, k)** shows the same but focused around the arcjet firing periods, while the right panel **(c, f, i, l)** shows the histograms of the differences between the adjusted GOES-18 values and the XGBoost (orange) and the current (blue) models.

In general, the corrected GOES-17 magnetic field measurements in the E component, obtained using the XGBoost algorithm (orange lines in Figures 4a, d, g, j), closely follow the adjusted GOES-18 values (green lines in Figures 4a, d, g, j). Additionally, the existing correction algorithm, which is based on a correction matrix, exhibits slight differences in long-term trends (blue lines in Figures 4a, d, g, j).

When zooming in on the arcjet firing periods each day, the XGBoost-based correction is observed to perform significantly better, particularly on July 26 (Figure 4e) and August 2, 2022 (Figure 4k). On July 22, while the existing correction algorithm removes the overall reduction in the magnetic field measurements during the arcjet firing period, a shoulder remains visible at 19:30 (Figure 4b). Conversely, the XGBoost-based correction algorithm eliminates the shoulder at the beginning of the arcjet firing but introduces a dip in the measurements (Figure 4b). On July 29, the XGBoost-based correction algorithm erroneously produces reduced values toward the end of the arcjet firing period, starting around 20:15 (Figure 4h).

We then compared the distributions of the differences between the adjusted GOES-18 magnetic field values in the E component and the corrections obtained using the XGBoost- and matrix-based algorithms (Figures 4c, f, i, l). In general, the XGBoost-based correction yields differences within 2 nT, whereas the existing correction algorithm can exceed 4 nT in some cases. This shows that the XGBoost algorithm provides better corrections overall.

**Figure 5:**
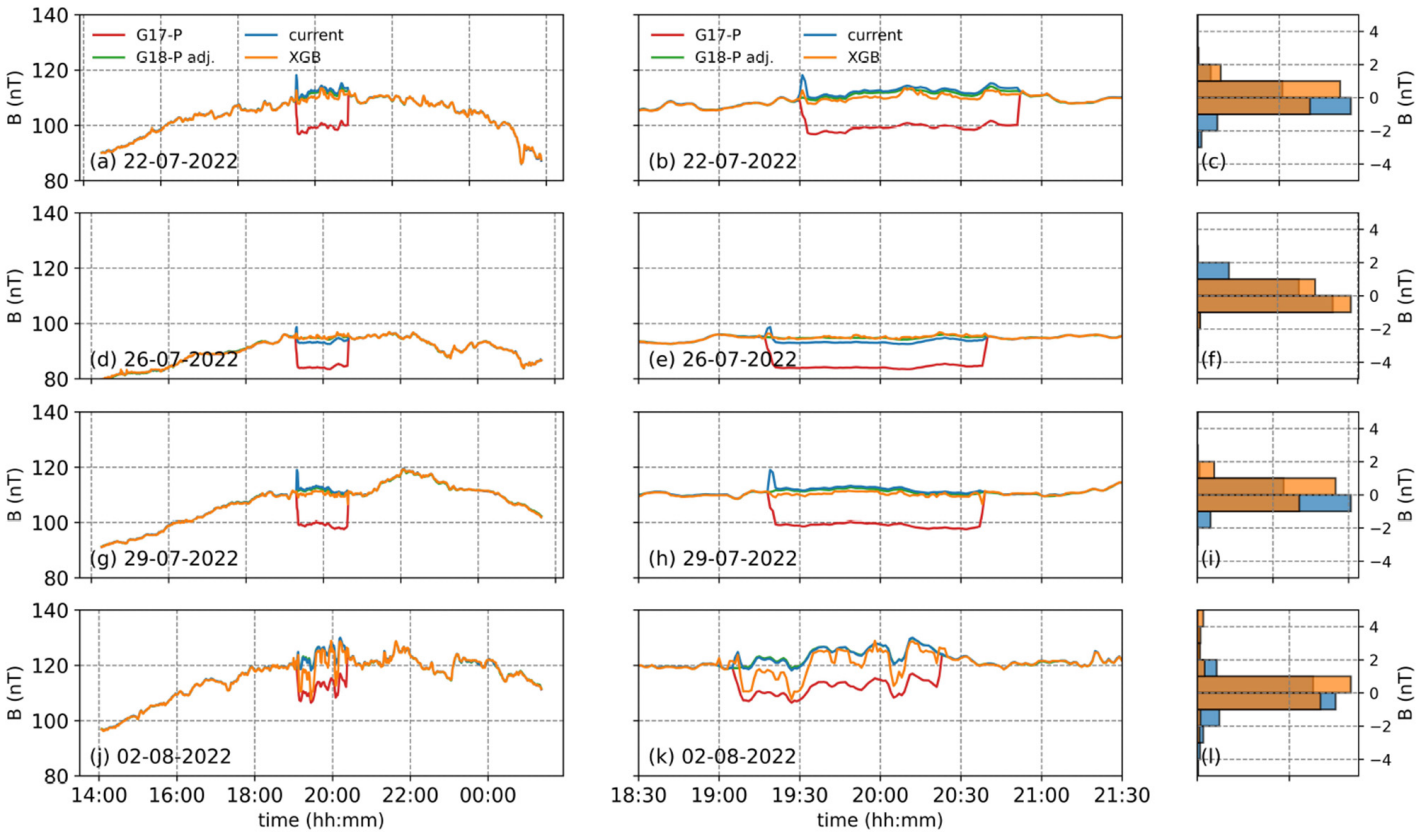
The same as Figure 4, but for the P component of the EPN coordinate frame. The left panel **(a, d, g, j)** shows 4 dates we tested the XGBoost developed to correct the P component of the EPN coordinate frame from GOES-17 (red), adjusted GOES-18 (green), the corrected GOES-17 using the current (blue) and XGBoost (orange) methods. The middle panel **(b, e, h, k)** shows the same but focused around the arcjet firing periods, while the right panel **(c, f, i, l)** shows the histograms of the differences between the adjusted GOES-18 values and the XGBoost (orange) and the current (blue) models.

Both the XGBoost-based algorithm and the existing correction method produce results without any deviation in the long-term trend of the P component (Figures 5a, d, g, j). However, sudden spikes at the beginning of nearly every arcjet firing event can be observed in the existing correction method (Figures 5a, d, g, j). When zooming in on the arcjet firing periods, these structures become more pronounced, whereas the XGBoost method does not generate spikes of comparable magnitude (Figures 5b, e, h, k). Additionally, the XGBoost correction algorithm fails to properly correct the arcjet contamination on August 2 (Figure 5k), which is also evident from the R^2^ and MSE values (Table 3).

Overall, the XGBoost correction algorithm generally performs slightly better than the existing correction algorithm for the P component (Figures 5c, f, i, l).

**Figure 6:**
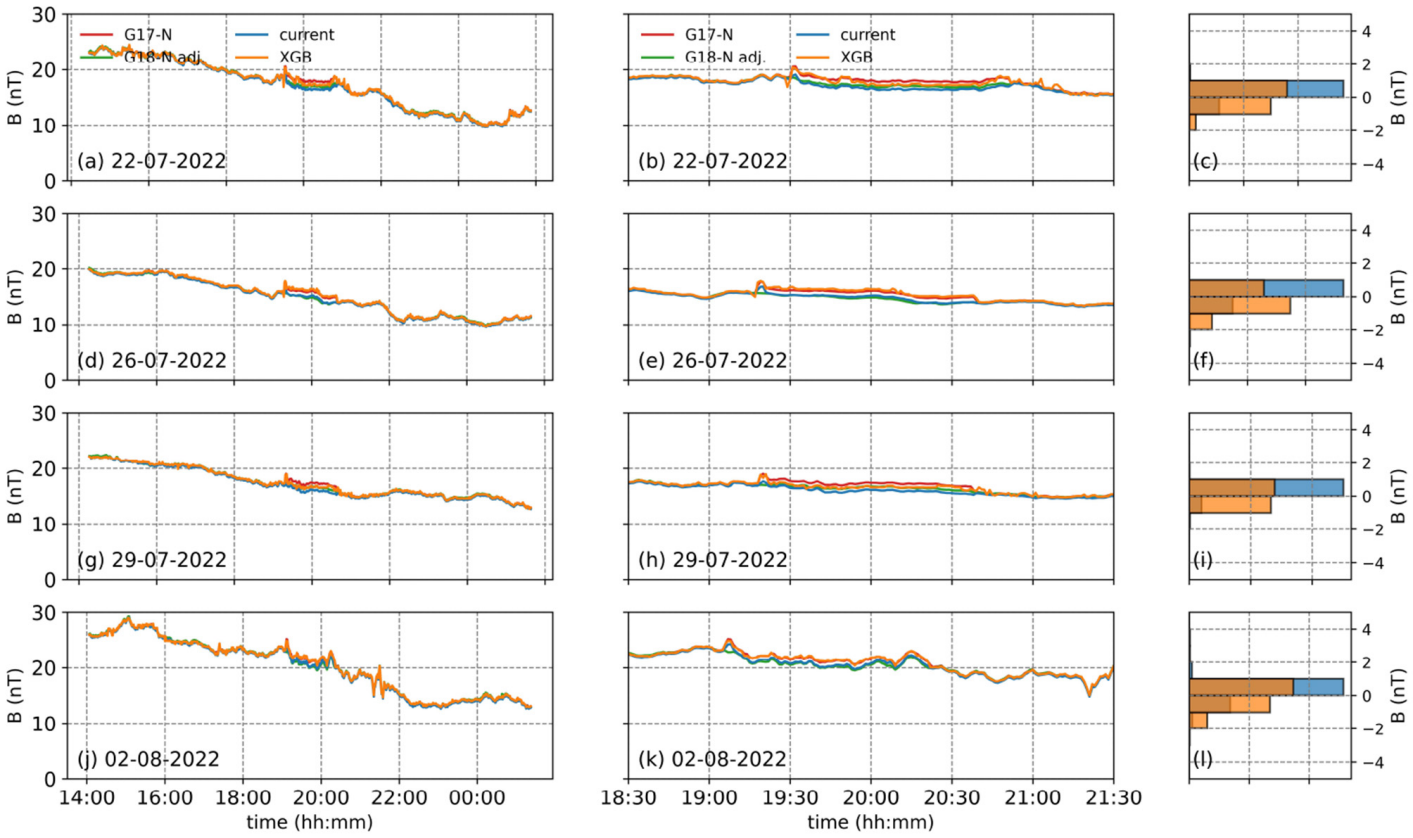
The same as Figure 4, but for the N component of the EPN coordinate frame. The left panel **(a, d, g, j)** shows 4 dates we tested the XGBoost developed to correct the N component of the EPN coordinate frame from GOES-17 (red), adjusted GOES-18 (green), the corrected GOES-17 using the current (blue) and XGBoost (orange) methods. The middle panel **(b, e, h, k)** shows the same but focused around the arcjet firing periods, while the right panel **(c, f, i, l)** shows the histograms of the differences between the adjusted GOES-18 values and the XGBoost (orange) and the current (blue) models.

For the N component, results from the test data indicate that the XGBoost correction algorithm slightly underperforms compared to the existing correction algorithm (Figure 6). Although the XGBoost algorithm provides corrections without any deviation from the long-term trend (Figures 6a, d, g, j), it does not correct the arcjet contamination as effectively as the existing correction algorithm when zoomed in Figures 6b, e, h, k.

This is also evident from the distributions of the differences between the adjusted GOES-18 magnetic field values in the N component and the corrections produced by the XGBoost and current methods (Figures 6c, f, i, l). The primary reason for this limitation is the insufficient amount of training data, particularly in the N component, which had fewer contaminated magnetic field measurements compared to the others. Unfortunately, the limited data availability is a direct consequence of the short collocation period.

### 3.1 Uncertainties and potential bias

In addition to the performance of the model, it is important to consider uncertainties at the instrument and platform levels that may influence the corrected results.

First, daily and seasonal changes in the thermal environment in geostationary orbit can cause diurnal structure and long-term drifts on the raw measurements, especially for the earlier R-series MAG sensors. Intersatellite analyses show that GOES-16 OB data exhibit artificial diurnal variations of order ±3 nT (1σ ≈ ±1.5 nT), whereas GOES-17 OB exhibits minimal daily variation and long-term stability within ~±2 nT (Rich et al., 2024). By contrast, the GOES-18 GMAG demonstrates much improved thermal stability: on-orbit OB-IB differences remain ≲±0.2 nT under diurnal cycling, and overall accuracy meets the NOAA ±1 nT requirement (excluding arcjet periods) (Loto'aniu et al., 2023).

Second, zero-level (offset) calibration and its refinement can introduce step-like biases if not updated. For GOES-17, yaw-flip maneuvers (180° rotations) were used to refine the OB zero offsets (e.g., −0.25 nT in P and +1.82 nT in N) and reduce long-term OB differences with other GOES spacecraft (Rich et al., 2024). Related analyses also indicate component-dependent biases on GOES-15 that manifest as pre/post-yaw-flip shifts (Rich et al., 2024). During the GOES-17/18 collocation, IB-OB statistics further show a mean offset in the GOES-17 N component (IB−OB ≈ −2.1 ± 0.62 nT), while GMAG (GOES-18) IB-OB means are near zero (Loto'aniu et al., 2023). Additionally, GOES-17 experienced a 2021 safehold event after which a small residual bias shift persisted (Loto'aniu et al., 2023).

Third, sensor placement and thermal coupling lead to IB/OB differences. Prior work showed the GOES-16 IB sensor is more thermally susceptible than OB, producing time-varying offsets of several nT tied to eclipse seasons and diurnal heating; ML corrections reduced these variations from ~3-5 nT to ~0-2 nT (E component) but some residual, MLT-dependent offsets remained (Inceoglu and Loto'aniu, 2021). These findings motivate our use of GOES-18 as the reference and our emphasis on spacecraft-intrinsic inputs for the correction.

In the context of our study, these factors imply that (i) residual uncertainty in the corrected GOES-17 series is bounded below by the stability/accuracy of the GOES-18 GMAG (order ~1 nT) and by any residual, component-dependent GOES-17 biases during the collocation; and (ii) IB and OB arcjet responses are highly similar in our data, but long-term IB/OB thermal susceptibilities differ. Practically, this is why we (a) trained against adjusted GOES-18 (collocation-aligned) values, (b) withheld OB data from test days, and (c) evaluate performance by component and day. We recommend users treat start/end-of-burn transients and days with known configuration changes or post-anomaly bias shifts with additional caution, and we view uncertainty quantification (e.g., confidence intervals on corrections or QC flags) as a natural extension for future operational use.

It should be noted that the XGBoost correction was designed to address plume-related arcjet contamination during firing periods and does not explicitly correct for the small residual thermal offsets (~1-2 nT) that can persist for hours after thruster shutdown (Califf et al., 2020a). These long-lived effects were removed during preprocessing and remain a separate calibration challenge.

## 4 Conclusions

The GOES magnetometers provide critical measurements for space weather monitoring and scientific research. However, the magnetic field data measured by the GOES missions are periodically contaminated by arcjet thruster firings due to attitude and maneuver corrections, introducing artificial disturbances that can impact both operational and research applications. The existing correction method, the correction matrix approach, mitigates these effects, but struggles with transient variations and residual errors, necessitating a more adaptive solution.

In this study, we developed an XGBoost-based machine learning model for each component of the EPN coordinate frame to correct arcjet-induced contamination in the GOES-17 magnetometer data using GOES-18 as a ground truth, after adjusting for longer-term trends. Our results demonstrate that the XGBoost shows great promise in reducing artificial disturbances, particularly in that it outperforms the existing correction in mitigating the non-linear features observed in the P component at the start and end of thruster firings. Although the model shows strong performance, some limitations remain due to training data constraints, particularly in certain magnetic field components in the EPN frame.

Although our model was developed and evaluated retrospectively, the underlying architecture and speed of XGBoost inference suggest that, with appropriate data preprocessing pipelines, the approach could be adapted for near-real-time implementation. This opens the possibility of future operational integration, enabling better correction of thruster-related contamination as part of the NOAA space weather monitoring workflow.

From an operational standpoint, interpretability remains a critical factor when assessing correction methods. Although the existing matrix approach is deterministic and familiar to operators, it can leave residual artifacts that resemble genuine geophysical events, such as magnetopause crossings, potentially leading to false positives. In contrast, ML-corrected data more effectively suppresses such artifacts but introduces a level of complexity in traceability and confidence, especially when corrections are based on patterns learned from other satellites. For operational users, a hybrid approach, in which ML corrections are applied but are accompanied by quality flags or confidence intervals, may offer the best balance between accuracy and interpretability.

We emphasize the importance of satellite collocation, such as the overlapping orbits of GOES-17 and GOES-18, as it provides a unique opportunity for cross-satellite calibration and validation, which is essential for improving magnetometer data accuracy and enhancing space weather monitoring capabilities.

## Data Availability

The training, validation, and test data, which were used for our models to correct arcjet-related anomalies in the magnetic field data, contain several different components. These include 1) magnetic field data in the EPN coordinate frame from two GOES-R series magnetometers, GOES-17 and GOES-18. And 2) arcjet specific data, including current and voltage supplied to the arcjets, and a binary arcjet flag derived from these values. Different components of these training data are subject to different export controlled restrictions. GOES-R Level 1b (L1b) data are generally publicly available on the NCEI product site (https://www.ncei.noaa.gov/products/goes-r-magnetometer). The L1b files contain the magnetic field data and the arcjet flag. However, the GOES-18 data from the time period used for this study were produced prior to provisional maturity validation of the data product, and as such are not available in the public archive. The GOES-17 and GOES-18 magnetic field data and the associated arcjet flag for days corresponding to the training, validation and test set will be published in a Zenodo repository (doi: 10.5281/zenodo.15721786) alongside the trained models. These data have been retroactively reprocessed, and have the same calibrations applied as the publicly archived GOES-R L1b data (temperature, zero-level offset and alignment). The arcjet flag is a binary flag indicating no arcjet firing (flag value 0) or arcjet firing (1). Thus, it is possible to identify the periods of arcjet firings in the magnetic field data. The arcjet currents and voltages, however, cannot be made available publicly due to International Traffic in Arms (ITAR) restrictions. The raw telemetry corresponding to currents and voltages is available in the Level 0 (L0, https://doi.org/doi:10.25921/sv2f-sm24) GOES-R MAG files which can be requested and evaluated on a case-by-case basis through NCEI customer support (ncei.info@noaa.gov). Please note that the specific process to convert L0 telemetry values into scientific units is not part of the L0 metadata, and the relevant documentation to do so cannot be released due to ITAR restrictions. Users may be able to create synthetic current and voltage estimates based on the information provided both in this paper, and the previous paper on the arcjet correction (Califf et al., 2020a). For further information or questions about the GOES-R data products, users can email swx.mag@noaa.gov.

## Footnotes

^1^As noted in the NOAA Transition to Operations notice: https://www.goes-r.gov/users/transitionToOperations17.html.
